# Supplementary material for: Data on Utility in Cost–Utility Analyses of Genetic Screen-and-Treat Strategies for Breast and Ovarian Cancer
Source: Cancers (Basel). 2021 Sep 29;13(19):4879. doi: 10.3390/cancers13194879 (PMC8508224; doi:10.3390/cancers13194879)
Supplement: Supplementary file 1 [file cancers-13-04879-s001.zip › cancers-1318340-supplementary-Table S1.pdf]

cancers MDPI

Supplementary Material

Data on Utility in Cost–Utility Analyses of Genetic Screen-and-Treat Strategies for Breast and Ovarian Cancer

Julia Simões Corrêa Galendi *, Vera Vennedey, Hannah Kentenich, Stephanie Stock and Dirk Müller

University of Cologne, Faculty of Medicine and University Hospital of Cologne, Institute of Health Economics and Clinical Epidemiology, Gleueler Str. 174-176, Cologne 50923, Germany.
* Correspondence: julia.simoes-correa-galendi@uk-koeln.de

Table S1. Sources of data on utility.

**Citations**	**Design**	**Instruments used**	**States on which data is provided (I-IV)**	**Cited by**
		Sources of Utilities		
Peasgood et al. (2010)	Systematic literature review of 13 databases	TTO, SG, EQ-5D, VAS	II (RRM)<br>III (BC)	Correa (III), Asphaug (II/III), NICE (III), Hurry (III), Sun (III), Moya-Alarcon (III), Müller (III), Eccleston, Li (III), Tuffaha (III)
Grann et al. (U.S., 2010)	Preference ratings of 243 respondents (83 with mutations, 160 controls)	TTO	I<br>II (RRM/RRSO)<br>III (BC/OC)	Correa (I/II), Hurry (I/II), NICE (I/II), Sun (II), Moya-Alarcon (II), Müller (I), Eccleston (I), Li (I)
Havrilesky et al. (U.S. 2009)	Preference ratings of 50 respondents from U.S. (13 with OC, 37 healthy controls)	VAS, TTO	III (OC)	Asphaug (III), Hurry (III), Sun (III), Moya-Alarcon (III), Müller (III), Tuffaha (III)
Grann et al. (U.S., 1999)	Preference ratings of 177 respondents (42 with mutations, 135 controls)	TTO	II (RRM/RRSO)<br>III (OC)	Kwon '19 (II), Müller (II), Li (II/III), Kwon '10 (II), Holland (II/III)
Griffith et al. (UK, 2004)	124 healthy women in Wales, UK, undergoing genetic assessment. Mean ratings for the self-rated health status scale of the EuroQol EQ-5D.	EQ-5D	II (RRM/RRSO)	NICE, Asphaug, Li
Lidgren et al. (Sweden 2007)	361 women included in a naturalistic cross-sectional observational study	TTO	III (BC)	Kwon '19, Kwon '10
Tengs et al. (U.S., 2000)	Systematic literature review of 3 databases and bibliographies of various review articles	TTO, SG	III (BC/OC)	Kwon '19, Kwon '10

Stein et al. (U.K., 2007)	66 women receiving chemotherapy for OC with results presented to a group of 38 members of the general population	EORTC QLQ-C30, SG	III (OC)	Correa
Geiger et al. (U.S., 2007)	Survey to 195 women with previous mastectomies and to a random sample of 117 untreated women at increased risk of BC	Modeled on or drawn directly from IES-R, CES-D, SF-36	IV	Holland
Lloyd et al. (UK, 2006)	Preference ratings of 100 healthy respondents from the UK (13 with OC, 37 healthy controls)	SG	III (BC)	Kwon '10
Connor-Spady et al. (U.S., 2005)	Questionnaires provided to 52 BC patients with poor prognosis at seven time points	FLIC, EQ-5D	III (BC)	Holland
Jansen et al. (U.S., 2004)	Questionnaires provided to 448 women with early-stage BC to assess their perception of freedom in choice of treatment and its consequences	VAS, EuroQol, HADS	III (BC)	Holland
Polsky et al. (U.S., 2002)	Preference ratings of women age 67 or older treated for localized BC	VAS, EuroQol, HUI	III (BC)	Holland
Capelli et al. (Canada, 2001)	Preference ratings of 169 respondents from Canada (60 with BC, 58 high-risk relatives of women with BC and 51 healthy controls)	SG	I	Holland
Grann et al. (U.S., 1998)	Preference ratings of 54 respondents (community-based healthy women)	TTO	II (RRSO)<br>III (BC/OC)	Tengs (II/III)
Unic et al. (NL, 1998)	Preference ratings of 54 women with various risk profiles for BC	TTO	II (RRM)	Tengs
Gerard et al. (Australia, 1993)	Validity study for an Australian cost-utility analysis with 180 women	TTO	II (RRM)	Tengs
de Haes et al. (NL, 1991)	Preference ratings from two samples (18 employees of the Department of Public Health and Social Medicine and 13 experts in BC)	VAS	II (RRM)<br>III (BC)	Tengs (II/III)
Justifications for assumptions				
Sie et al. (NL, 2016)	One-year follow-up surveys for 108 BC patients evaluating long-term experiences (i.e., satisfaction and psychological distress)	GHQ-12, IES-her, QoL, BC worry, risk perception	Justification for the assumption that no disutility should be attributed to genetic testing	Moya-Alarcon, Eccleston
Halbert et al. (U.S. 2011)	Observational study to estimate the long-term impact of genetic testing for BRCA1/2 mutations in 167 women	MICRA	Justification for the assumption that no disutility should be attributed to genetic testing	Moya-Alarcon, Eccleston
Lerman et Al: (U.S., 1996)	Interviews at baseline and 1-month follow-up of male and female members	CES-D, targeted scales	Justification for the assumption	Holland

	(n=279) of families with BRCA1-linked hereditary BC/OC	for functioning and well-being	that there is increased utility of a negative test result	
Beran et al. (U.S., 2008)	Trajectory of psychological status in 155 women at risk of BC and OC prior to undergoing genetic testing through 1 year later	CES-D, IES-R	Justification for the assumption that disutility should be attributed to genetic testing but resolved within one year.	Eccleston
van Oostrom et al. (NL, 2003)	Questionnaire to 65 female participants (23 carriers, 42 non-carriers) and interviews of 51 women five years after genetic test disclosure	HADS, IES, CWS	Justification that disutility of genetic testing persisted for 5y	Holland

BC = breast cancer, CWS = Cancer Worry Scale, CES-D = Center for Epidemiologic Studies – Depression Scale, EQ-5D = EuroQol, FLIC = Functional Living Index-Cancer, GHQ = General Health Questionnaire, HADS = Hospital Anxiety and Depression Scale, HUI = Health Utilities Index, IES = Impact of Event Scale, IES-her = heredity-specific psychological distress, IES-R = Impact of Events Scale, OC = ovarian cancer, QoL = Quality of Life, SG = Standard Gamble, MICRA = Multidimensional Impact of Cancer Risk Assessment, TTO = Time Trade off.
